# Giving volume to the elephant in the imaging room

**DOI:** 10.15252/emmm.202216876

**Published:** 2022-11-02

**Authors:** Anurag Agrawal, Disha Agrawal

**Affiliations:** ^1^ Trivedi School of Biosciences Ashoka University Sonepat India; ^2^ Maulana Azad Medical College Delhi University Delhi India

**Keywords:** Respiratory System

## Abstract

Rudolf Virchow, the founder of cellular pathology, held that even when physical or chemical investigations yield the laws of physiology or medicine, the anatomist can still proudly state: This is the structure in which the law becomes manifest. In his words, “physiology presupposes anatomy.” Pathological anatomy studies, at usual microstructural scales (approximately 1–100 μm), via light microscopic 2D histology, provided many insights into structure–function relationships of health and disease. For example, such studies established the progression of granulomas, bronchial erosions, microcavities, and destructive lung disease in tuberculosis. While histologic studies remain the cornerstone of such efforts, the advent of nano or micro‐X‐ray computed tomography (n/μCT) has now made it additionally possible to obtain 3D visualizations of soft and hard tissues, while preserving the tissue for additional investigations. This has applications for old as well as new diseases (Katsamenis *et al*, 2019; Tanabe & Hirai, 2021).

## Graphical abstract

A. Agrawal and D. Agrawal discuss the study by A. Steyn and colleagues in the last issue of *EMBO Mol Med*. That reports high‐resolution 3D atlas tuberculous and COVID‐19 lung lesions established using micro‐X‐ray computed tomography.

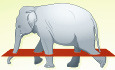

In the last issue of *EMBO Molecular Medicine*, Wells *et al* conducted extensive n/μCT‐based investigations into the spectrum of tuberculous or COVID‐19 lesions, contextualizing histological and clinical radiological findings in the same patient (Wells *et al*, 2022). In tuberculosis, this followed up on previous work by them and the Africa Health Research Institute showing that conventional histology alone underestimates the complexity of pathologic lesions or their connections with physiologic pulmonary structures (Dartois & Dick, 2021; Wells *et al*, 2021). Tubercular granulomas were found to be complex branching tube‐like structures relatable to bronchial dissemination and obliterated airways. This is in contrast to the common spherical depiction of granulomas, perhaps due to extrapolations from circular 2D cross‐sections on classical histology (Fig 1). The work has literally added a new dimension to our understanding of tubercular granulomas.

**Figure 1:**
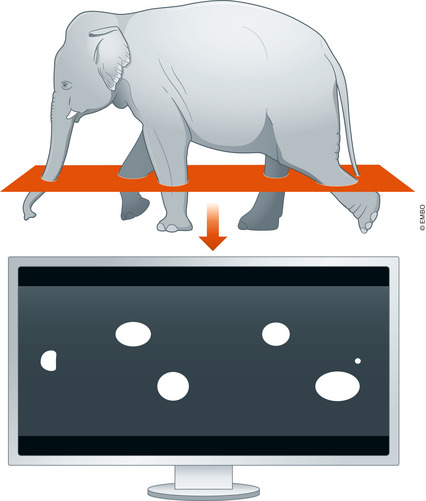
The elephant in the imaging room Volumetric n/μCT of intact tissue can reveal unrecognized important complexity missed during 2D histological sectioning.

Imaging of autopsy specimens from severe COVID‐19 patients provided important imaging correlates for poorly understood clinical findings. Severe COVID‐19 does not always have the typical pathophysiological features of acute respiratory distress syndrome, despite a general radiological similarity and a pneumonic etiology (Gattinoni *et al*, 2020; Mahjoub *et al*, 2020). Pulmonary vascular thrombosis has been suggested as a likely additional factor causing disproportionate hypoxemia (McFadyen *et al*, 2020). Here, the clear delineation of vascular tree‐in‐bud sign, with dilated tortuous peripheral microvasculature and focal hemorrhage, strongly supports thrombotic microangiopathy as part of the COVID‐19 pathophysiology. Using maximum intensity projections, it was possible to directly visualize organizing, cylindrical vascular thrombus with early canalization at the site of adherence to the vessel wall. The ability to contextualize vascular pathology within the larger lung architecture, while visualizing the micro‐architecture in unprecedented detail, is what makes n/μCT a valuable tool. Again, there is a dimensional difference between seeing small circular or spherical occlusive thrombi on histology versus noting long organizing cylindrical branching thrombi, while considering the potential benefit of anti‐thrombotic therapy in severe COVID‐19 patients (Leentjens *et al*, 2021). Such evidence, if available earlier in the course of the pandemic would have certainly been very helpful in determining optimal therapeutic management.

The atlas of n/μCT imaging of tuberculous and COVID‐19 lungs is an excellent introduction to the possibilities being opened by new radiologic imaging methods. As always, there is no perfect method, and it is important to consider the relative advantages of each. For example, paraffin‐embedded fixed tissue samples remain difficult (but not impossible) to study due to similar density of paraffin and tissue. Preserving complex tissue architecture while snap‐freezing or fixing specimens remains an art. Density resolution between very different soft tissues may be poor. Indeed, it was necessary for Wells *et al* to perform a variety of studies, including classical histology, to contextually interpret n/μCT findings. Fine ultrastructural or molecular pathological anatomy remains beyond the scope of radiology for now, but soon, with greater availability of equipment and methods, a nondestructive n/μCT 3D scan with virtual dissection may routinely precede and guide final sectioning and staining.
